# Immunohistochemical Evaluation of Ki-67 Proliferation Marker in Various Biological Variants of Ameloblastoma

**DOI:** 10.7759/cureus.99979

**Published:** 2025-12-23

**Authors:** Gaurav Salunkhe, Anu S Issac, Rohit Jha, Deepak Kolte, Padma P Datla, Rahul VC Tiwari, Heena Dixit, Seema Gupta

**Affiliations:** 1 Department of Oral Pathology, Dr GD Pol Foundation's Yerala Medical Trust Dental College and Hospital, Navi Mumbai, IND; 2 Department of Pathology, Dr. Somervell Memorial CSI Medical College and Hospital, Thiruvananthapuram, IND; 3 Department of Oncology, Rajendra Institute of Medical Sciences, Ranchi, IND; 4 Department of Oral and Maxillofacial Surgery, Bharati Vidyapeeth Dental College and Hospital, Navi Mumbai, IND; 5 Department of Oral and Maxillofacial Surgery, GSR Institute of Craniofacial Surgery, Hyderabad, IND; 6 Department of Oral and Maxillofacial Surgery, RKDF Dental College and Hospital, Sarvepalli Radhakrishnan University, Bhopal, IND; 7 Department of Blood Cell, Commisionerate of Health and Family Welfare, Hyderabad, IND; 8 Department of Orthodontics, Kothiwal Dental College and Research Centre, Moradabad, IND

**Keywords:** ameloblastoma, biological variation, immunohistochemistry, ki-67 antigen, proliferation

## Abstract

Introduction

Ameloblastoma remains one of the most enigmatic and locally destructive benign odontogenic tumors, posing significant therapeutic challenges owing to its variable biological behavior across histopathological subtypes. The present study aimed to evaluate and compare the proliferative activity using Ki-67 labelling index (LI) among solid/multicystic ameloblastoma (SMA), unicystic ameloblastoma (UA), and desmoplastic ameloblastoma (DA), and to correlate the findings with their known clinical aggressiveness and recurrence potential.

Materials and methods

Thirty archival formalin-fixed, paraffin-embedded blocks of ameloblastoma were retrieved. The ameloblastoma cases comprised 12 SMA (predominant plexiform pattern), 10 UA, and 08 DA cases. Four or five micrometer sections were subjected to immunohistochemical staining using a ready-to-use mouse monoclonal anti-Ki-67 antibody. Three blinded oral pathologists independently counted Ki-67-positive tumor nuclei in three hot spot high-power fields (×100), and the labelling index was calculated as the percentage of positive nuclei per 1,000 tumor cells. Data were analyzed using one-way analysis of variance (ANOVA) and Tukey’s post-hoc test; p <0.05 was considered significant.

Results

Ki-67 LI was significantly higher in SMA (mean 75.44 ± 18.58%, range 20.56-93.89%), followed by UA (22.81 ± 4.81%), and lowest in the DA (11.7 ± 15.44%; p=0.001). Post-hoc analysis confirmed significantly higher expression in SMA than in UA (p=0.001) and DA (p=0.001), with no significant difference between UA and DA (p=0.353). Excellent interobserver agreement was achieved (ICC=0.99).

Conclusion

SMA exhibited markedly higher proliferative activity than UA and DA, explaining their greater local aggressiveness and recurrence risk. The Ki-67 LI is a reliable and reproducible immunohistochemical marker that can aid risk stratification, treatment planning, and prognostic prediction in ameloblastoma.

## Introduction

Odontogenic tumors encompass a diverse spectrum of lesions originating from odontogenic residues, including the odontogenic epithelium and ectomesenchyme, often mimicking the sequential development of normal tooth formation. They range from benign neoplasms and hamartomas to malignant entities, with ameloblastoma representing a classic example of an odontogenic neoplasm [1]. Ameloblastoma is a benign yet locally aggressive tumor arising from enamel organ-type tissue without enamel formation. Its pathogenesis remains controversial, potentially stemming from remnants of the dental lamina, Hertwig's sheath, epithelial cell rests of Malassez, odontogenic cysts, such as dentigerous cysts, or genetic mutations in tooth development cells [2].

Clinically, it predominantly affects the mandibular molar-ramus region in the third to seventh decades, showing no sex predilection, but a higher incidence in certain ethnic groups. Symptoms include painless swelling, facial deformity, malocclusion, and rare extensions to the sinuses or nasal floor. Radiographically, it presents as uni- or multilocular radiolucencies with "soap bubble" or "honeycomb" appearances, which are often associated with unerupted teeth. Classified into solid/multicystic (SMA), unicystic (UA), and peripheral types, SMA is the most common (86%) and aggressive, with higher recurrence rates, while UA is less invasive unless it shows mural invasion [3-5].

Histopathologically, variants include follicular, plexiform, acanthomatous, granular cell, basal cell, and desmoplastic patterns, with follicular and plexiform, each comprising approximately one-third of the cases. Although ameloblastomas are histologically benign, their locally aggressive and invasive nature presents a striking paradox, with rare instances of metastasis reported in the literature [3,6]. Cell proliferation, a hallmark of neoplasia, is regulated by the cell cycle and can be assessed using markers such as Ki-67, a nuclear protein expressed in active phases (G1, S, G2, and M) but absent in resting (G0) cells [7]. Ki-67 helps quantify growth fractions, correlates with tumor aggressiveness and recurrence, and provides prognostic insights for treatment planning. This study aimed to demonstrate, correlate, and compare Ki-67 expression in SMA, UA, and desmoplastic ameloblastoma variants with their clinical behavior. The primary objectives of the present study were to assess the proliferative activity of SMA, UA, and desmoplastic ameloblastoma by evaluating the Ki-67 labelling index using immunohistochemistry, to quantitatively compare Ki-67 expression among these histopathological variants, and to determine inter-observer reliability in the assessment of Ki-67 labelling index by independent oral pathologists. The secondary objectives were to correlate Ki-67 labelling index with the known biological behavior and aggressiveness of the different ameloblastoma variants, to evaluate the association between Ki-67 expression and clinicodemographic variables such as age, sex, site, and jaw involvement, and to assess the potential utility of Ki-67 labelling index as a prognostic indicator for biological aggressiveness, recurrence risk, and treatment planning in ameloblastoma.

## Materials and methods

This laboratory-based observational comparative immunohistochemical study was conducted in the Department of Oral Pathology, Dr. DY Patil Dental College and Hospital, Nerul, Navi Mumbai, for six months in 2014, after obtaining approval from the Institutional Ethics Committee (DYPUNM/IEC/2014/Th-OP-02). Thirty formalin-fixed paraffin-embedded tissue blocks previously diagnosed by senior oral pathologists were retrieved from the departmental archives. These comprised 30 histologically confirmed cases of ameloblastoma, subdivided into three histopathological variants (a predominant plexiform pattern, unicystic ameloblastoma, and desmoplastic ameloblastoma).

From each paraffin block, two serial sections of 4 or 5 µm thickness were cut using a semi-automatic rotary microtome (Leica RM 2245, Germany). One section was stained with Hematoxylin and Eosin to confirm the original diagnosis, whereas the second was mounted on poly-L-lysine-coated slides (BioGenex Lab, California, USA) for immunohistochemical staining. Deparaffinization was carried out by heating the slides on a slide warming table at 65°C for 30 min, followed by two changes of xylene (seven minutes each) and descending grades of alcohol, after which the sections were brought to water. Antigen retrieval was performed using freshly prepared tris(hydroxymethyl)aminomethane-ethylenediaminetetraacetic (Tris-EDTA) buffer (pH 6.0) (1.21 g Tris base and 0.37 g EDTA in 1000 mL distilled water) and subjected to heat-induced epitope retrieval in an EZ-Retriever microwave system (Biogenex, USA) at 96°C for two cycles of 10 minutes each, followed by cooling to room temperature and rinsing in phosphate-buffered saline (PBS, pH 7.4).

Endogenous peroxidase activity was blocked by incubating the sections with peroxidase block (Biogenex) for 15 minutes. Non-specific protein binding was prevented by applying Power Block (Biogenex) for 10 min. The sections were then incubated with a ready-to-use mouse monoclonal anti-Ki-67 antibody (clone MIB-1, Biogenex, USA) for 60 min at room temperature in a humid chamber. This was followed sequentially by Super Enhancer (Biogenex) for 20 min and Polymer-conjugated Horseradish Peroxidase reagent (Poly-HRP) reagent (Biogenex) for 30 min. The antigen-antibody reaction was visualized using freshly prepared 3,3′-diaminobenzidine tetrahydrochloride (DAB) chromogen as the substrate, yielding a brown nuclear precipitate. Sections were counterstained with Harris hematoxylin, dehydrated through ascending grades of alcohol, cleared in xylene, and mounted with Distrene, Plasticiser, and Xylene (DPX). 

Immunostained slides were examined under a binocular light microscope (BX43; Olympus Corp., Tokyo, Japan) by three independent oral pathologists blinded to the histopathological subtype. At 100x magnification, three fields exhibiting the highest density of positively stained nuclei (hotspots) were selected. Positive cells were counted in each hotspot at 100x magnification (high-power field). Hot-spot selection at ×100 magnification was performed to identify three fields with the highest density of positively stained nuclei, followed by counting at ×400 magnification. This approach is widely recommended for heterogeneous tumors such as ameloblastoma, as it captures biologically relevant proliferative zones (often peripheral ameloblast-like cells in plexiform patterns) and prevents underestimation of the labelling index (LI) in areas of low proliferation. The Ki-67 LI was calculated as the percentage of positively stained tumor cell nuclei out of 1000 tumor cells counted per case [8].

\[ \text{LI} = \left( \frac{\text{Ki-67+ nuclei}}{1000} \right) \times 100 \]

**Figure 1:**
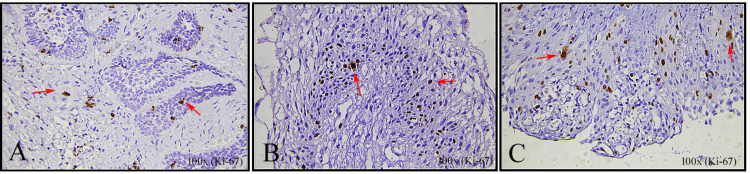
Photomicrographs of Ki-67 immunohistochemical staining (100x magnification) in different histological variants of ameloblastoma showing proliferating cells (brown nuclear staining, indicated by red arrows) (A) Desmoplastic ameloblastoma, (B) Solid multicystic ameloblastoma, and (C) Unicystic ameloblastoma. Original images of the samples from the study.

Interobserver variability was minimized by taking the mean of the three observers' counts for the final statistical analysis (Figure 1).

Statistical analysis

Data were analyzed using the Statistical Package for Social Sciences (SPSS) version 25.0 (IBM Inc., Armonk, New York, USA). Demographic data were presented as frequency and percentage for categorical variables and as mean and standard deviation for continuous variables. Data normality was assessed using the Shapiro-Wilk test (p>0.05 for all groups, confirming no significant deviation from normality) and homogeneity of variances via Levene's test (p=0.412, indicating equal variances), while independence of observations was ensured through the retrospective selection of distinct archival cases without related sampling. Interclass correlation (ICC) between the observers' scores was performed. The intergroup comparison of the percentage of mean LI of Ki-67 in different histopathological types of ameloblastoma was performed using one-way analysis of variance (ANOVA) followed by post-hoc Tukey’s test. Statistical significance was set at p<0.05.

## Results

Table 1 shows the demographic profiles of 30 patients with ameloblastoma. The sample consisted of 20 (66.7%) males and 10 (33.33% females, with a male-to-female ratio of 2:1. The mandible was affected in 28 (93.3%) cases compared to the maxilla in two (6.7%) cases, and the posterior region was the most common site. Histologically, the solid/multicystic ameloblastoma (SMA) type was most frequently found in 12 (40%), followed by unicystic ameloblastoma (UA) in 10 (33.3%), and desmoplastic in eight (26.7%) cases. These findings align with the established pattern of ameloblastoma, showing male and mandibular posterior predominance.

**Table 1:** Demographic and clinicopathological characteristics of 30 patients with ameloblastoma Values are expressed as frequency (n) and percentage (%)

Parameters	Category	Frequency (n)	%
Sex	Male	20	66.7%
	Female	10	33.3%
Jaw	Mandible	28	93.3%
	Maxilla	2	6.7%
Site	Posterior	23	76.7%
	Anterior	7	23.3%
Histopathological type of ameloblastoma	Solid multicystic	12	40%
	Unicystic	10	33.33%
	Desmoplastic	8	26.67%

Table 2 demonstrates the excellent inter-observer reliability of the Ki-67 LI assessment [8]. The ICC value was 0.99 (95% CI: 0.99-1.00), with statistical significance (p=0.001). This near-perfect agreement confirmed the high reproducibility and consistency between observers, validating the robustness and reliability of the Ki-67 quantification method used in the study.

**Table 2:** Intraclass correlation coefficient (ICC) for Ki-67 labelling index [8] among three independent observers *p=0.001 denotes statistical significance; CI = confidence interval

Intra class correlation coefficient	Lower 95%-CI	Upper 95%-CI	F stats	p-value
1	0.99	1	638.75	0.001*

Table 3 reveals that Ki-67 LI, a marker of proliferative activity, was significantly highest in SMA (mean 75.44 ± 18.58%, range 20.56-93.89%), followed by UA (22.81 ± 4.81%) and lowest in desmoplastic type (11.7 ± 15.44%). The mean age at presentation was similar across the subtypes (SMA: 32.33 years; desmoplastic: 32.5 years; UA: 29.1 years). The markedly elevated Ki-67 expression in SMA indicates higher proliferative potential and biological aggressiveness compared to UA and desmoplastic variants, supporting its more aggressive clinical behavior and higher recurrence risk.

**Table 3:** Age at diagnosis and Ki-67 labelling index [8] according to histopathological subtype of ameloblastoma (n=30) Data are presented as mean ± standard deviation (SD)

Parameter	Histopathological type	Minimum	Maximum	95% Confidence interval for mean	Mean ± SD
Labelling index of Ki-67 (%)	Solid multicystic	20.56	93.89	63.63 - 87.24	75.44 ± 18.58
	Desmoplastic	0	46.22	-1.22 - 24.61	11.7 ± 15.44
	Unicystic	18.11	33.44	19.37 - 26.25	22.81 ± 4.81
Age (years)	Solid multicystic	6	69	20.4 - 44.27	32.33 ± 18.78
	Desmoplastic	27	42	28.43 - 36.57	32.5 ± 4.87
	Unicystic	9	55	18.11 - 40.09	29.1 ± 15.37

Table 4 shows that age did not differ significantly among ameloblastoma histopathological types (p=0.854). In contrast, Ki-67 LI revealed highly significant intergroup variation (p=0.001). This confirms that SMA exhibits markedly higher proliferative activity than UA and desmoplastic subtypes, explaining its greater biological aggressiveness and higher recurrence potential.

**Table 4:** Comparison of age and Ki-67 labelling index [8] among ameloblastoma subtypes using one-way analysis of variance (ANOVA) *p=0.001 denotes statistical significance; df - degree of freedom

Parameters	Sum of squares	df	Mean square	F stats	p-value
Age	72.73	2	36.37	0.16	0.854
Labelling index	24408.98	2	12204.49	58.07	0.001*

Tukey's post-hoc analysis confirmed that SMA showed significantly higher Ki-67 expression than the desmoplastic (mean difference=63.74%, p=0.001) and UA (mean difference=52.63%, p=0.001) subtypes. No significant differences were found between the desmoplastic and UA types (p=0.353). These findings established that the SMA variant is the most proliferative and biologically aggressive subtype of ameloblastoma (Table 5).

**Table 5:** Post-hoc pairwise comparisons of Ki-67 labelling index [8] between ameloblastoma subtypes (Tukey's honestly significant difference test) *p=0.001 denotes statistical significance; CI - confidence interval

Pairwise groups	Mean difference	Standard error	T stats	p-value	95% CI lower limit	95% CI upper limit
Solid multicystic - desmoplastic	63.74	6.62	9.63	0.001*	46.85	80.63
Solid multicystic - unicystic	52.63	6.21	8.48	0.001*	36.78	68.47
Desmoplastic - unicystic	-11.11	6.88	-1.62	0.353	-28.67	6.44

## Discussion

The present study investigated the proliferative activity of ameloblastoma variants, such as SMA, UA, and desmoplastic ameloblastoma, through Ki-67 immunohistochemical expression, aiming to correlate these findings with their clinical behavior, aggressiveness, and recurrence potential. The results demonstrated significant differences in the Ki-67 LI among the variants, with SMA exhibiting the highest proliferative activity, followed by UA and desmoplastic ameloblastoma. These findings align with the established clinical behavior of these variants and provide insights into their biological aggressiveness, supporting the use of Ki-67 as a prognostic marker for ameloblastoma management.

The mean Ki-67 LI of 75.44% in SMA is higher than most previously reported values. This elevated index likely reflects several methodological factors: (i) exclusive inclusion of SMA cases with predominant plexiform pattern, known for robust peripheral proliferation in ameloblast-like cells; (ii) strict hotspot selection at ×100 magnification to identify maximal staining density; (iii) counting of 1,000 tumor epithelial nuclei per case at ×400; and (iv) use of a highly sensitive ready-to-use MIB-1 antibody.

The Ki-67 antigen is predominantly expressed during the latter stages of the G1 phase of the cell cycle, while cells in a quiescent state (G0 phase) exhibit a complete absence of Ki-67 expression. Because of its nonexistence in quiescent cells (G0 phase), Ki-67 protein has been extensively utilized as a significant prognostic biomarker for tumors [9]. The significantly higher Ki-67 LI in SMA underscores its aggressive nature, characterized by local invasiveness and a high recurrence rate, often reported between 15-29% [6]. The comparative risk of recurrence was observed to be 3.15 times higher when conservative management was employed for primary SMA, in contrast to the implementation of radical treatment [10]. In contrast, a previous study reported no correlation between Ki-67 levels and the aggressiveness of ameloblastoma [11].

The elevated proliferative activity in the SMA, particularly in the plexiform pattern, is consistent with the results of previous studies. For instance, Sandra et al. [12] reported higher Ki-67 expression in SMA than in UA, which was attributed to increased cellular turnover in the tumor's epithelial component. Follicular and plexiform patterns, predominantly in the SMA, are known to exhibit robust cellular proliferation due to their complex architecture and active stromal-epithelial interactions [5]. The present study's findings of a mean LI of 75.44% in the SMA corroborate these observations, suggesting that the high proliferative index contributes to its invasive growth into the surrounding bone, leading to the characteristic "soap bubble" radiographic appearance and higher recurrence risk.

In contrast, UA displayed a lower Ki-67 LI, reflecting its less aggressive behavior and better prognosis. UA is often associated with a cystic architecture and a lower recurrence rate (10-20%) when treated with enucleation, particularly in cases without mural invasion [13]. Our investigation indicated that the mean Ki-67 LI was calculated to be 22.81 ± 4.81%, which is similar to the findings documented by Bologna-Molina et al. [14], who reported it to be 15.28%. The reduced proliferative activity in UA aligns with studies by Sandra et al. [11], who noted lower Ki-67 expression in UA than in SMA, correlating with its slower growth and less invasive nature. However, UA cases with mural invasion may exhibit higher proliferative activity, approaching SMA levels, which warrants further investigation in larger cohorts to stratify UA subtypes based on Ki-67 expression [15]. The UA cases in the current study were not subclassified according to mural invasion, which may account for the moderate LI observed.

Desmoplastic ameloblastoma exhibited the lowest Ki-67 LI (11.7 ± 15.44%), consistent with its distinct histopathological and clinical profile. Characterized by extensive stromal desmoplasia and compressed epithelial islands, this variant is less invasive but poses diagnostic challenges owing to its atypical radiographic presentation, often resembling fibro-osseous lesions [16]. The low proliferative activity observed in this study aligns with the findings of Bologna-Molina et al. [14], who reported minimal Ki-67 expression in desmoplastic ameloblastoma, suggesting a slower growth rate and lower recurrence potential compared to SMA. The unique stromal environment of the desmoplastic variant may suppress epithelial proliferation, thereby contributing to reduced Ki-67 expression.

The lack of significant age differences across variants (p = 0.854) suggests that proliferative activity, rather than patient demographics, drives the biological behavior of ameloblastomas. The male predominance (2:1) and mandibular posterior predilection in this study are consistent with the global epidemiological data, reinforcing the representativeness of the sample [2,3,17]. The excellent interobserver reliability validates the robustness of the Ki-67 quantification method, ensuring reproducibility and reliability for clinical applications.

The significant intergroup variation in Ki-67 LI, confirmed by Tukey's post-hoc analysis, highlights SMA's distinct proliferative profile compared with UA and desmoplastic variants. The absence of significant differences between UA and desmoplastic ameloblastoma suggests that these variants share less aggressive biological behavior despite their histopathological differences. These findings are supported by Yoon et al. [18], who demonstrated that Ki-67 expression correlates with recurrence risk, with higher expression in ameloblastic carcinoma.

Clinical implications

Ki-67 LI may provide additional insight into the biological behavior of ameloblastoma variants and can serve as an adjunctive tool alongside conventional clinicopathological and radiographic assessment. Elevated expression in SMA aligns with its known greater aggressiveness, whereas lower values in unicystic and desmoplastic variants are consistent with their relatively indolent behavior. However, Ki-67 should not be used in isolation to guide decisions regarding radical versus conservative surgery. Treatment planning must remain multidisciplinary and incorporate established factors such as tumor variant, size, radiographic extent, and presence of mural invasion in unicystic cases.

Limitations

The present study had several limitations. The retrospective design and relatively small sample size may limit generalizability, particularly for rare variants, such as desmoplastic ameloblastoma. The lack of subclassification of UA cases by mural invasion may have obscured the variations in proliferative activity within this group. Additionally, the study focused solely on Ki-67, and incorporating other proliferative markers (such as proliferating cell nuclear antigen as PCNA and minichromosome maintenance protein-2 as MCM-2) could provide a more comprehensive assessment of tumor behavior. Long-term clinical follow-up data on recurrence were not included, limiting the ability to directly correlate the Ki-67 LI with clinical outcomes. Finally, the study was conducted at a single institution, potentially introducing a selection bias.

## Conclusions

The present study revealed significant variation in Ki-67 expression among histopathological variants of ameloblastoma, with the solid/multicystic type showing the highest proliferative activity, followed by unicystic and desmoplastic variants. These findings underscore biological differentiation in proliferative potential across subtypes and align with their differing degrees of local aggressiveness. While Ki-67 labelling index provides insight into tumor cell proliferation and may aid in characterizing biological behavior, it should not be regarded as a definitive marker for prognostic stratification. Comprehensive assessment of ameloblastoma aggressiveness requires integration of multiple clinicopathological features, radiographic findings, and potentially additional molecular markers. Future prospective studies with larger cohorts and longitudinal data are warranted to further elucidate the clinical relevance of proliferative markers in this heterogeneous odontogenic tumor.
